# A rare case of De Garengeot hernia: CT findings

**DOI:** 10.1093/bjrcr/uaae009

**Published:** 2024-02-29

**Authors:** Maria Iovino, Anna Chiara D’Elia, Maurizio Rispo, Alfonso Rispo, Arturo Brunetti, Fabio Sandomenico

**Affiliations:** Radiology Unit, San Giuliano Hospital, Giugliano In Campania, Naples 80014, Italy; Department of Diagnostic Imaging and Radiotherapy, University Federico II, Naples 80100, Italy; Radiology Unit, San Giuliano Hospital, Giugliano In Campania, Naples 80014, Italy; Surgery Unit, San Giuliano Hospital, Giugliano In Campania, Naples 80014, Italy; Department of Diagnostic Imaging and Radiotherapy, University Federico II, Naples 80100, Italy; Radiology Unit, Buon Consiglio Fatebenefratelli Hospital, Naples 80123, Italy

**Keywords:** computed tomography, appendix, appendicitis, hernia, femoral, De Garengeot hernia, hernia, inguinal, Amyand hernia

## Abstract

We report a case of “De Garengeot’s hernia” (DGH), a rare condition that occurs when the inflamed appendix is localized inside a femoral hernia. The appendix may be involved in inflammatory or necrotic processes and the treatment is emergency surgery. It is usually discovered by chance during surgery. It occurs in 0.5%-5% of all femoral hernias. In 0.08%-0.13% of cases, the appendix can present inflammatory or necrotic processes due to the narrowness of the neck of the femoral canal; in these cases, an emergency surgery is required through a no standard surgical procedure. In the other cases, it is usually found accidentally during surgical repair of the hernia or more rarely diagnosed preoperatively by CT. Therefore, the purpose of our study is to report a case of DGH describing CT main findings in order to improve the preoperative diagnosis.

## Clinical presentation

A 72-year-old man presented to the emergency department of our institution with a painful right-sided groin mass. On physical examination, the vital signs were normal. Laboratory evaluation revealed an increase of white blood cells and inflammatory indices. Ultrasound imaging was performed and showed a right-sided groin mass containing an intestinal loop suspected to be the vermiform appendix, surrounded by a hyperechoic periappendiceal fat without fluid collection.

**Figure 1.**
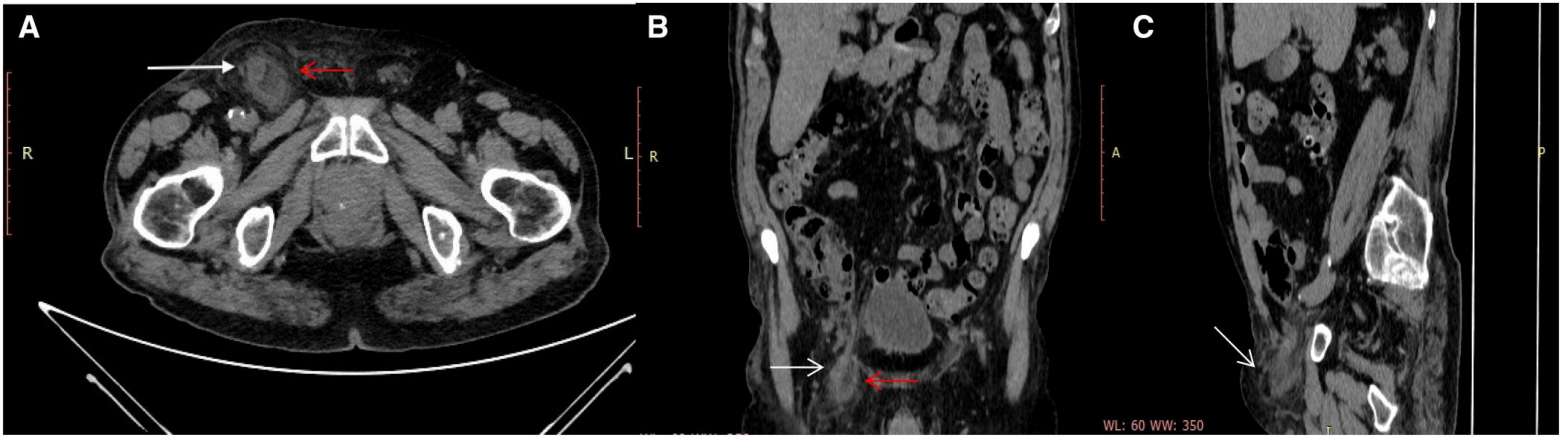
(A) CT axial scan of the abdomen shows dilated appendix (white arrow) in right femoral hernia (red arrow) with signs of inflammation of the surrounding fat. (B) MPR coronal reconstruction shows the appendix (white arrow) as a tubular structure extending from the caecal base within a right femoral hernia (red arrow), (C) MPR sagittal reconstruction shows the extension of the right hernia sac within the femoral (white arrow).

We performed Helical CT scan obtained without intra-venous iodinated contrast agent for allergy of patient to contrast agents in a non-deferrable emergency. CT images well demonstrated an appendix extending into a right femoral hernia with thickening of surrounding omental fat, well depicted in reformatted multiplanar (MPR) sagittal and coronal images (Figure 1A-C).

## Differential diagnosis

The differential diagnosis of DGH should include Amyand hernia. “Amyand’s hernia” is defined by the presence of appendix within an inguinal hernia, due to the surgeon who described this condition for the first time.^1^

Even in this case, CT can play an important role in the differential diagnosis. Indeed, the CT findings that can differentiate femoral hernias from inguinal hernias are the relationship between the hernia sac and pubic tubercle and the venous compression.

When the hernia sac is located medial to the pubic tubercle, the diagnosis of inguinal hernia can be made with confidence.^2^ If the hernia sac extends lateral to pubic tubercle and compresses the femoral vein due to the narrowness of the femoral canal it is likely to be a femoral hernia.

## Treatment

The treatment was emergency surgery.

**Figure 2.**
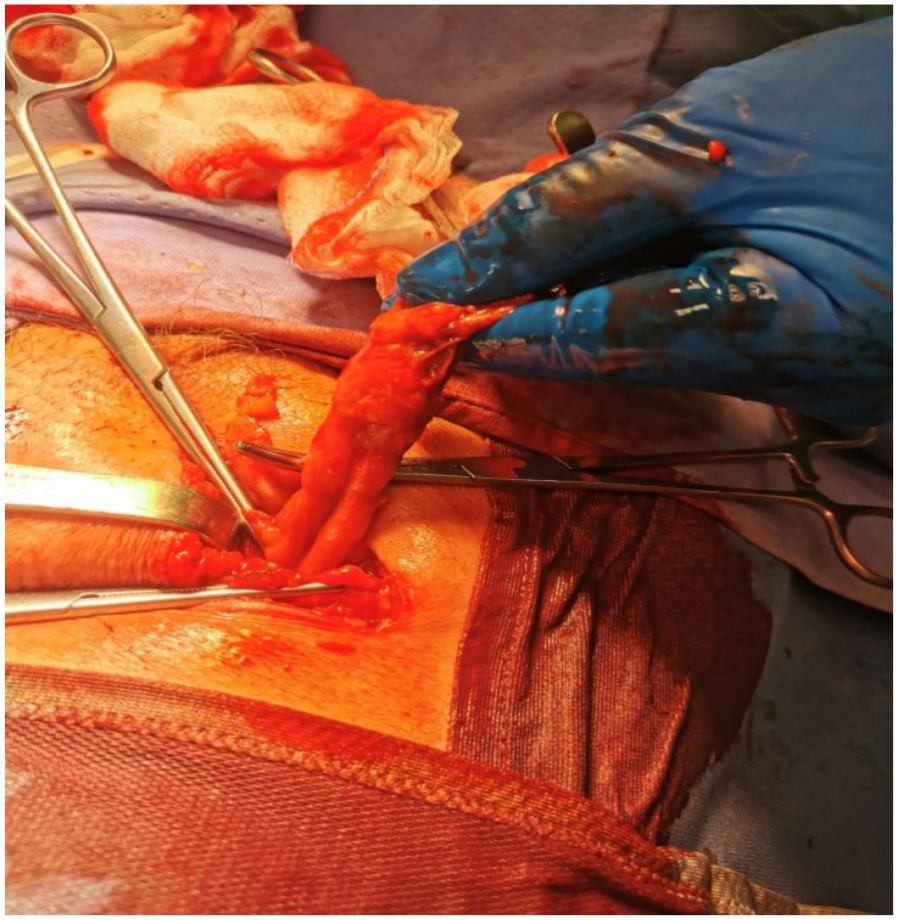
A femoral hernia containing the appendix during surgery through an open approach.

An open approach was chosen: the hernia sac was opened through a right crural incision; the vermiform appendix and the omental fat were found inside (Figure 2). Therefore, an appendectomy was performed and the hernia sac was closed. The hernia defect was repaired using a polypropylene mesh plug (Lichtenstein technique). The appendectomy and hernia repair were successfully completed without complications.

Post-operative recovery was uneventful and the patient was discharged after few days.

## Discussion

Femoral hernias are abdomen hernias that pass through the femoral ring into the femoral canal, posterior and inferior to the inguinal ligament and medial to the femoral vessels.^3^ Instead, inguinal hernias occur above the inguinal ligament and through the inguinal canal; they can be direct hernias when pass medially to the inferior epigastric vessels or indirect hernias when originate lateral to the inferior epigastric vessels.^4^

The incidence of abdominal hernias is about 5% in the world population and in 80% of these cases they are inguinal hernias; in only 5% they are femoral hernias.^4^

The contents of the sac can include viscera, omentum, or others abdominal structures. Rarely, the sac may contain vermiform appendix.

Predisposing factors for appendix herniation are an abnormal anatomical position during embryological development and a large caecum.^5^

The appendix can be involved in inflammatory processes when obstruction occurs at the hernial neck and vessel compression leads to bacterial overgrowth.^6^

Femoral hernia that contains appendix is called “De Garengeot’s hernia”.

The term “De Garengeot’s hernia” (DGH) is referred to a rare condition in which the appendix is localized inside a femoral hernia. It is named after Rene Jacques Croissant De Garengeot, the surgeon who first reported a case of femoral hernia containing the appendix.^7^

DGH can be classified into five classes according to the gross appearance of the appendix.^8^

It can be linked to other clinical conditions that lead to abdominal wall weakness or increase intra-abdominal pressure.^9^ Therefore the female gender is the most important risk factor due to the body changes during pregnancy.

The clinical presentation of DGH is usually a painful groin mass with associated leucocytosis and elevated PCR. It can mimic an incarcereted or stranguleted femoral hernia; therefore most DGH are diagnosed intraoperativily.

CT can play an important role in achieving preoperative diagnosis: DGH should be considered when caecum is in a low position with a blind-ended tubular structure within the hernia sac and stranding of nearby fat with high sensibility and specificity.^10^

In conclusion, even though DGH is a rare condition and still a challenge for the radiologist, preoperative diagnosis has to be improved in order to increase preoperative diagnosis and indicate the correct management of the patients.

## Learning point

- The term “De Garengeot’s Hernia” (DGH) is referred to a rare condition in which the appendix is localized inside a femoral hernia. The appendix may be involved in inflammatory or necrotic processes.
- CT can play an important role in the differential diagnosis.
- The treatment is surgery.
- The differential diagnosis of DGH should include Amyand hernia which is defined by the presence of appendix within an inguinal hernia.
